# Characterization of colorectal mucus using infrared spectroscopy: a potential target for bowel cancer screening and diagnosis

**DOI:** 10.1038/s41374-020-0418-3

**Published:** 2020-03-20

**Authors:** Jayakrupakar Nallala, Charles Jeynes, Sarah Saunders, Neil Smart, Gavin Lloyd, Leah Riley, Debbie Salmon, Nick Stone

**Affiliations:** 1 Biomedical Physics, School of Physics and Astronomy, University of Exeter, Exeter, EX4 4QL UK; 2 Living Systems Institute, University of Exeter, Exeter, EX4 4QD UK; 3 Cellular Pathology Department, Royal Devon & Exeter Hospital, Exeter, EX2 5AD UK; 4 Department of Surgery, Royal Devon and Exeter Hospital, Exeter, EX2 5DW UK; 5 Phenome Centre Birmingham, University of Birmingham, Birmingham, B15 2TT UK; 6 Biocatalysis Centre, Biosciences, University of Exeter, Exeter, EX4 4QD UK

**Keywords:** Biomarkers, Gastrointestinal cancer

## Abstract

Biological materials presenting early signs of cancer would be beneficial for cancer screening/diagnosis. In this respect, the suitability of potentially exploiting mucus in colorectal cancer was tested using infrared spectroscopy in combination with statistical modeling. Twenty-six paraffinized colon tissue biopsy sections containing mucus regions from 20 individuals (10 normal and 16 cancerous) were measured using mid-infrared spectroscopic imaging. A digital de-paraffinization, followed by cluster analysis driven digital color-coded multi-staining segmented the infrared images into various histopathological features such as epithelium, connective tissue, stroma, and mucus regions within the tissue sections. Principal component analysis followed by supervised linear discriminant analysis was carried out on pure mucus and epithelial spectra from normal and cancerous regions of the tissue. For the mucus-based classification, a sensitivity of 96%, a specificity of 83%, and an area under the curve performance of 95% was obtained. For the epithelial tissue-based classification, a sensitivity of 72%, a specificity of 88%, and an area under the curve performance of 89% was obtained. The mucus spectral profiles further showed contributions indicative of glycans including that of sialic acid changes between these pathology groups. The study demonstrates that infrared spectroscopic analysis of mucus discriminates colorectal cancers with high sensitivity. This concept could be exploited to develop screening/diagnostic approaches complementary to histopathology.

## Abstract (web summary)

Mucus was tested as a potential biological material for screening/early diagnosis of colorectal cancer using infrared spectroscopy. Based on a digital histopathology and statistical modeling approach, cancerous and non-cancerous samples were classified with an area under the curve performance of 95% based on mucus spectral profiles, indicating changes in the glycan component of mucins.

## Introduction

Colorectal cancer is the fourth most common type of cancer in the UK and one of the most common types in the Western world. The incidence is highest in the older population and since the 1990s, colorectal cancers have shown a stable incidence rate. Over half of these cases are diagnosed at later stage [1]. Currently, screening is the best option to triage individuals at risk of developing colorectal cancer; early diagnosis being the best prevention strategy.

In the UK, colorectal cancer screening is offered via Fecal Immunochemical Test and Bowel Scope test, also called the flexible sigmoidoscopy [2]. Suspicious lesions mostly in the form of polyps are endoscopically removed and analyzed via histopathology, which is the current confirmatory test for cancer diagnosis.

In histopathology, changes in cell and tissue architecture are examined at microscopic level, which are the manifestations of much smaller-scale biomolecular changes such as those of nucleotides, proteins, sugars etc. Although, microscopic changes are clearly diagnosed using histopathology, in some cases, early molecular changes that are not yet expressed phenotypically are difficult to detect at the microscopic level. Immunohistochemistry is adjunctively used in histopathology to examine the expression levels of specific biomolecules in a semiquantitative manner; this usually needs multiple tissue sections for labeling different biomolecules of interest [3]. Combining these approaches, it is possible to obtain information on the holistic biomolecular changes, however patient waiting time and cost effectiveness have to be compromised. Furthermore, routine microscopic examination of large tissue samples is a laborious task that is time consuming and can be susceptible to observer bias.

In this regard, there are two important unmet clinical challenges that could potentially be improved in the process of cancer diagnosis:

1. Examining alternative biological materials for detecting early biomolecular changes.
2. Using novel analytical streams which could be coupled to multivariate statistical and machine learning approaches to digitize, automate, and thereby speed-up the process of cancer diagnosis in an objective manner.

For the latter, several studies have demonstrated the suitability of infrared (IR) spectroscopy approaches to probe and identify biomolecular changes in various types of cancer to detect and in some cases predict cancers [3–8].

IR spectroscopy is a non-destructive, label-free photonic technology that probes the distinct vibrational bonds of biomolecules such as DNA, RNA, proteins, lipids and sugars and provides a biochemical fingerprint of the measured material. The structural and compositional changes of the samples can be deciphered from the spectral peak shifts, varied peak intensities, and peak areas, using suitable analytical approaches. This capability of IR spectroscopy hence presents an important prospect to develop novel screening/diagnostic tools that could be made compatible with, and complementary to the current clinical diagnosis processes.

Using this approach, biological materials mostly comprising of tissue biopsies and cells have been tested, primarily with the aim to integrate IR spectroscopy into routine clinical diagnosis process [9–14]. Bio-fluids such as serum and sputum which are easy-to-obtain and less invasive compared with tissue biopsy procedures have also been tested using this approach for cancer diagnosis [15–17]. However, the complexity and the unknown nature of biomolecular constituents in bio-fluids pose significant challenges in identifying specific markers that could be used in cancer diagnosis. Other analytical techniques such as nuclear magnetic resonance and mass spectrometry (MS) have been used to target known potential markers for cancer diagnosis. However, rendering the techniques compatible enough to complement the existing clinical diagnosis process is another challenge [18, 19].

Hence, alternative biological materials that present early signs of cancer before the morphological expression at microscopic level could be hugely beneficial in cancer diagnosis.

Mucus, the viscous fluid covering the surface epithelia of organs, is known to undergo structural and quantitative biomolecular changes during cancer; especially the glycoprotein mucin, which is the predominant component in mucus [20]. Structurally, mucin glycoproteins comprise of a protein backbone onto which glycans are attached. Initially N-acetylgalactosamine (GalNAc) is attached onto specific amino acid residues on the protein which is then elongated by stepwise addition of monosaccharides such as N-acetylglucosamine (GlcNAc), galactose, mannose, and fucose in different combinations, and the chain is terminated by attachment of a sialic acid monomer. The glycans mediate diverse roles in functioning of the body. In comparison with normal glycans, cancer-associated abnormal glycans are known to have differential glycosylation patterns and are differentially sialylated and influence functions such as adhesion, tumor migration, and immunity [20–24]. Identification of cancer-associated changes in glycans is an important step in detecting early signs of cancer.

IR spectroscopy with its ability to probe the molecular composition of the sample that can be combined with novel multivariate and machine learning approaches could be a good candidate to detect these changes rapidly and in a label-free manner.

In view of this, a proof-of-concept study has been undertaken with the aim to test and identify biomolecular changes between normal and cancerous mucus present in colon tissue biopsies using IR spectroscopy. In the long term, the objective would be to test mucus, either collected as part of the FOBT, or from close to a suspected lesion under endoscopic examination with the aim to identify early signs of cancer.

## Materials and methods

Twenty-six formalin-fixed paraffin-embedded colon tissue biopsy samples (10 normal and 16 cancerous) from 20 individuals were obtained for this study. Fourteen samples (whole sections) from eight individuals were obtained from participants recruited to the Risk Stratification for Rectal Cancer Treatment (RIST) pilot project, via the Royal Devon and Exeter Tissue Bank (RDETB) in an anonymized manner. This is an ethically approved Tissue Bank (REC no: 16/SC/0162) set up to proactively collect and store “spare” tissue, and associated clinical data, available from routine clinical procedures for forth-coming studies examining disease specific biomarkers. THE RDETB is facilitated through the NIHR Exeter Clinical Research Facility. The 12 other samples from 12 individuals were obtained in the form of tissue micro array (TMA) from US Biomax, Inc. Each of the samples was sliced into 7-µm thin sections and placed on a barium fluoride (BaF_2_) slide (Crystran, UK) compatible with IR imaging. Sample details and the pathology information can be found in supporting information Table 1.

Hyperspectral imaging was carried out on these tissue sections to map mucus areas present within and close to normal and cancerous epithelial regions using an FTIR spectroscopic imaging system (Agilent 620 FTIR microscope coupled with an Agilent 670 FTIR spectrometer, Australia). The IR imaging set up uses a Globar® light source emitting mid-IR radiation which is then focused onto the 7-µm thick tissue section using a cassegrain condenser. The transmitted light was collected with a matched cassegrain objective and imaged onto a 128 × 128 pixel Focal Plane Array imaging detector to capture the IR image. Using ×15 cassegrain objectives a 700 × 700  µm^2^ sample area (field of view) is captured in a single tile made up of 5.5 × 5.5 µm^2^ pixels (at the sample). Several tiles were measured in mosaic to cover the region of interest on the tissues.

The set-up records all the frequencies (1000–3800 cm^−^^1^) at the same time and the output is an IR hyperspectral image where *x* and *y* axes contain the image coordinates and the *z* axis contain the IR spectral intensity values for each wavenumber, all obtained in the same image. Images were recorded in the mid-IR spectral range of 2–12 µm corresponding to 1000–3800 wavenumbers, at a spectral resolution of 8 cm^−1^.

For each tissue section, an adjacent 3-µm thick section was also obtained and stained with haematoxylin and eosin (H&E) for use as positive controls. These sections were examined by an expert histopathologist for marking regions of interest and to validate the spectro-morphological findings. Glycan standards (Sigma Aldrich, UK) in the form of powders were prepared as KBr pellets for IR transmission measurements. For this, 0.1 g of standard and 0.99 g of dry KBr powder were mixed in a mortar and pestle and pressed into thin discs using a 15 ton hydraulic press (Specac® UK). The pellets were immediately measured using the same parameters as that of the tissue images.

A total of 26 images were generated from 26 samples making a database of 1,622,016 spectra at a mean of 67,584 spectra/sample. From this, 24 samples showed epithelial regions and 20 samples showed mucus regions and were divided accordingly into two sets for further analysis.

### Spectral preprocessing

**Fig. 1:**
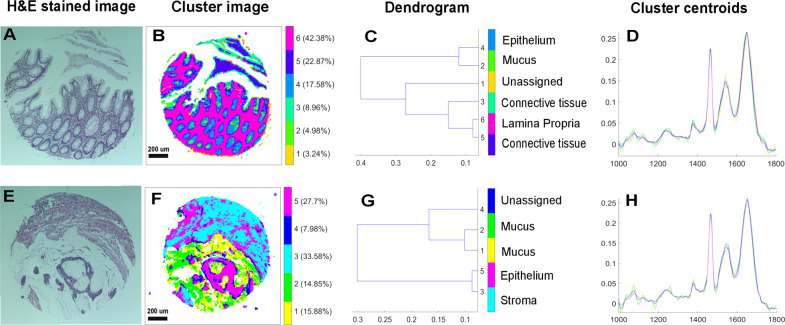
Segmentation of infrared spectral tissue images based on cluster analysis. Representative examples of a normal (**b**) and a cancerous (**f**) colon tissue segmented into respective histological features using six and five clusters, respectively, using HE stained images (**a**, **e**) as morphological reference. The corresponding dendrogram (**c**, **g**) representing the heterogeneity of the clusters and the cluster centroids (**d**, **h**) are also shown.

The IR images were acquired directly from FFPE tissues without any chemical de-paraffinization, with the intention to retain the mucus intact within the tissue. Alternatively, a digital de-paraffinization procedure based on Extended Multiplicative Signal Correction (EMSC) algorithm was used to neutralize the paraffin contributions together with other spectral interferences [25–28]. The digital de-paraffinization model based on a modified EMSC algorithm consists of an ‘interference matrix’ which is a separately measured pure paraffin spectral image that is incorporated into the EMSC algorithm. A fit analysis of this pure paraffin and the tissue spectra are performed against a ‘target spectrum’ or a ‘reference spectrum’ which is the mean spectrum of a representative tissue image that is commonly used for all the samples. A good fit indicates presence of pure ‘paraffin dominated’ regions which are then localized using the image coordinates and eliminated from the data analysis. These eliminated paraffin regions could be identified in the cluster images as white pixels (Fig. 1b, f). The paraffin present in the ‘tissue dominated’ regions is normalized and its influence on the spectral data is minimized. In addition to limiting the intra-sample variability due to paraffin, the use of a single common target spectrum for all samples also avoids the inter-sample variability by taking into account only the biochemical variability and not the baseline or paraffin variability across samples. A more detailed explanation of development and application of this approach could be found elsewhere in the literature [25–28]. In brief, spectra were preprocessed using EMSC to retain biomolecular variance from the tissue and eliminate undesired physical variance (e.g. paraffin, scattering artefacts) that could influence data classification. In addition to the digital de-paraffinization, the algorithm also performs baseline correction (4th order polynomial), smoothing (Savitzky-Golay 3rd order polynomial and 11 point fitting) and normalization needed for further spectral analysis.

### Cluster analysis

The preprocessed spectra of each IR image were independently subjected to k-means clustering. This process is an unsupervised algorithm that segments an image into pre-assigned class numbers based on the spectral variability [29]. The number of groups explored ranged from 3 to 11. By visually comparing the segmented images to the corresponding HE images and using pathologist’s validation, most representative class number was retained for further analysis.

### Multivariate data analysis

Principal component (PC) fed linear discriminant analysis (LDA), a supervised algorithm was used to separate the spectral features and classify the samples. This was carried out based on a leave-one-sample-out-cross-validation where each time a sample is taken out of the model and used as the test set while the remaining data set is used as training set with pathology labels. Principal component analysis (PCA) is a data reduction algorithm that reduces large spectral data sets to a smaller number of orthogonal variables called the principal components or principal component loadings. This removes collinear variables from the analysis. After computing the PCs, Analysis of Variance (ANOVA) was used to select the PCs up to a maximum of 25, showing a significant difference between the pathology groups at 99% significance level, to be used as inputs for LDA cross-validation [30]. The test sample was then projected onto the model and its predicted pathology classified. This was repeated until all the samples had been independently tested.

## Results

### Tissue segmentation

The segmentation results of representative normal and cancerous spectral images using cluster analysis are shown in Fig. 1. In Fig. 1b, a normal colon tissue section is segmented using six clusters sufficient enough to identify the important histological features based on the biomolecular fingerprint. Using the H&E image as reference (Fig. 1a) and pathologist’s validation, the clusters are assigned to the following histological groups: cluster 2-mucus, 4-epithelium, 3, 5-connective tissue, and 6-lamina propria and randomly color-coded for easy visualization. A small percentage of pixels represented by cluster 1 are not assigned to any histological class and appear to represent edge artefacts of the tissue. The histological assignment is reinforced based on the heterogeneity in the dendrogram (Fig. 1c) showing the biomolecular similarity (and dissimilarity), and also based on the spectral centroids (Fig. 1d) which for e.g. show typical mucus spectral features in the 1000–1300 cm^−1^ region [31]. In Fig. 1f, a cancerous colon tissue is segmented using five clusters, sufficient to identify the key histological features in comparison with the reference H&E image (Fig. 1e), which in this case are clusters 1, 2-mucus, 5-epithelium, and 3-stroma. The cluster 4 is not assigned to any histological class and appears to represent edge artefacts between the mucus and the epithelium. The heterogeneity of the histological groups is seen in the dendrogram (Fig. 1g) and the spectral features in the cluster centroids (Fig. 1h).

### Pathology classification

**Fig. 2:**
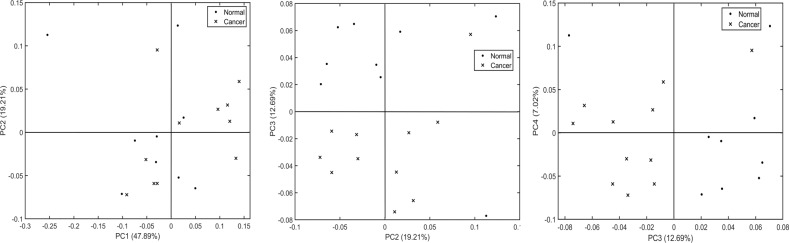
Principal component analysis scatter plot of normal vs. cancer groups using mean spectra of mucus. Normal and cancer group separation using principal components PC1 vs. PC2 (left), PC2 vs. PC3 (center) and PC3 vs. PC4 (right). The explained variance of each component is shown in brackets adjacent to the principal component number.

Following the cluster analysis, in order to identify the potential hidden biomolecular signatures of mucus indicative of carcinogenesis, a statistical modeling approach based on PCA-LDA was used. Firstly, the aim was to see if the mucus signatures in the normal tissues are different from that found in the cancerous tissues. For this, spectra corresponding to the mucus class were extracted from the cluster maps of each sample and PC scores were computed from the mean spectra using PCA as shown in Fig. 2. In the 2D scatter plot of the first four PCs, PC3 showed the best separation between normal and cancer samples (in both PC2 vs. PC3 and PC3 vs. PC4), with only one normal and one cancerous sample misclassified into the opposite group. The remaining PCs did not show any separation.

**Fig. 3:**
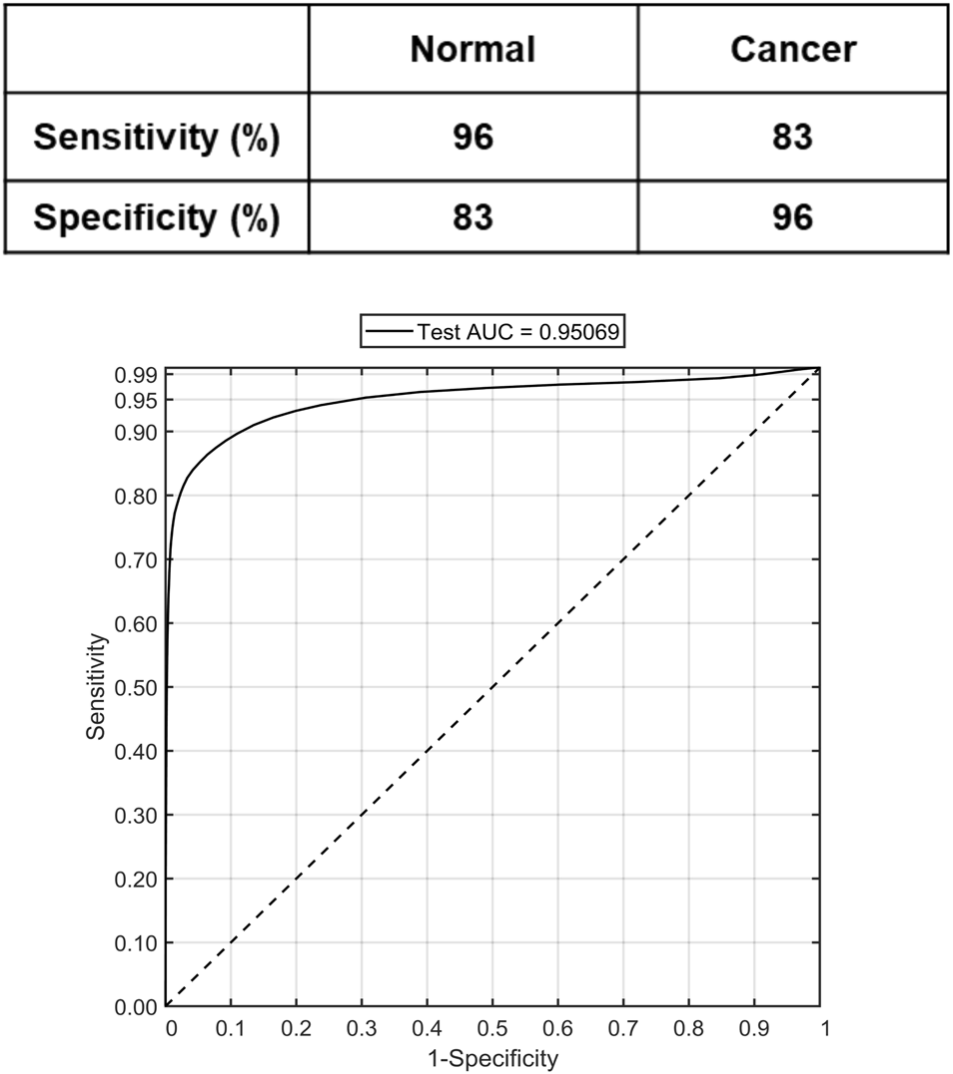
Classification performance of normal vs. cancer group using leave-one-sample-out cross-validation of mucus spectra based on principal component analysis followed by linear discriminant analysis. (Top) Sensitivity and specificity values for normal vs. cancer pathology model based on mucus spectra. (Bottom) Receiver operating characteristic (ROC) curves representing the mucus classification performance.

Building on this evidence of separation using mean spectra, the next aim was to see if PC fed LDA model, with mucus spectra as the input, could classify the samples into benign or malignant groups. For this, PCs were computed from all the mucus spectra extracted from the cluster maps. Following this, the scores of ANOVA selected PCs showing significant difference between the pathology groups up to a maximum of 25 PCs, at 99% significance level were used as inputs for an LDA classification model. In addition, Receiver operating characteristics (ROC) curves were plotted to evaluate the performance of the classifier. As shown in Fig. 3 (top panel), a sensitivity of 96%, a specificity of 83%, and an area under curve (AUC) value of 95% (bottom panel) was obtained for the leave-one-sample-out cross-validated classification model.

**Fig. 4:**
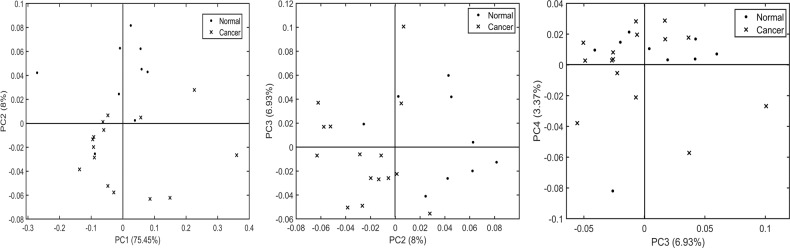
Principal component analysis scatter plot of normal vs. cancer groups using mean spectra of epithelium. Normal and cancer group separation using principal components PC1 vs. PC2 (left), PC2 vs. PC3 (center) and PC3 vs. PC4 (right). The explained variance of each component is shown in brackets adjacent to the principal component number.

**Fig. 5:**
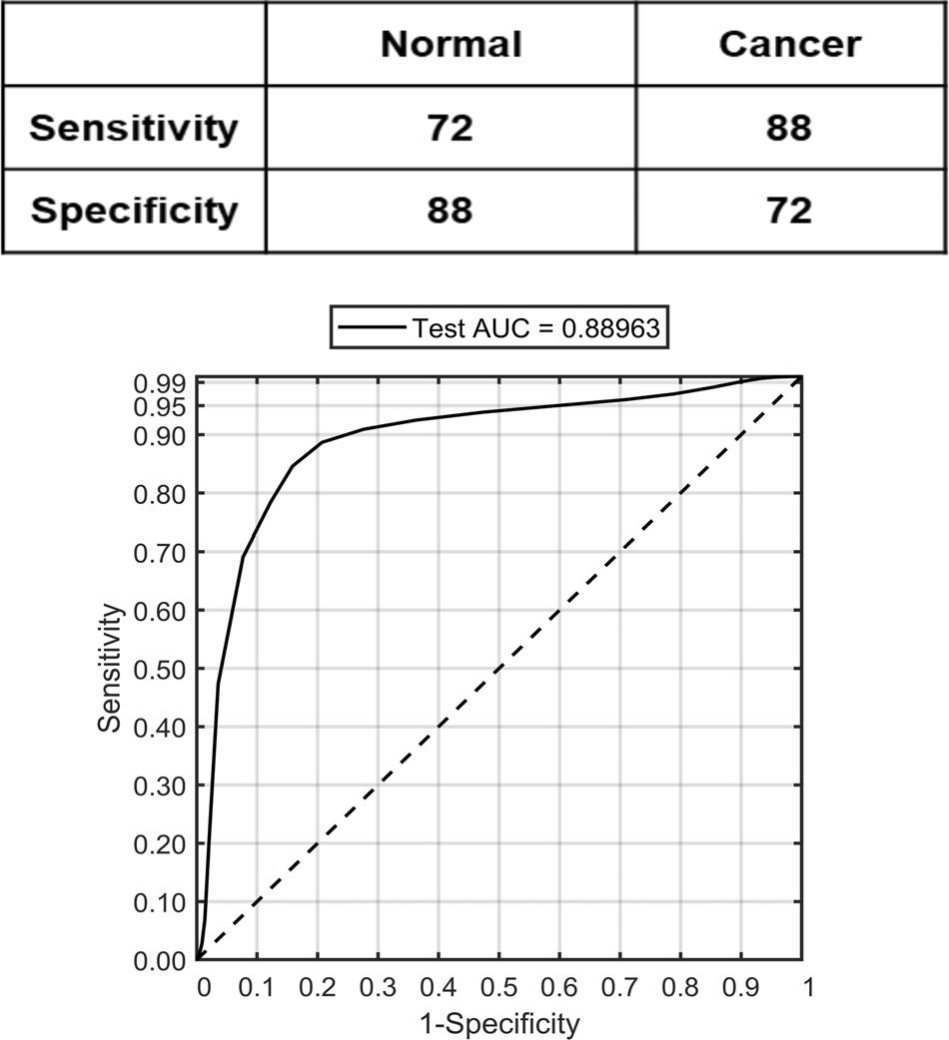
Classification performance of normal vs. cancer group using leave-one-sample-out cross-validation of epithelial spectra based on principal component analysis followed by linear discriminant analysis. (Top) Sensitivity and specificity values of the model for normal vs. cancer based on epithelial spectra. (Bottom) Receiver operating characteristic (ROC) curves representing the epithelium classification performance.

To evaluate the performance, mucus-based spectral classification was then compared with a similar classification analysis on epithelial spectra from the same samples. Comparing the PC scores from the mean epithelial spectra in Fig. 4, PC2 showed the best separation between normal and cancer samples (in both PC1 vs. PC2 and PC2 vs. PC3), with one normal sample misclassified into the cancer group and four cancerous samples misclassified into the normal group. The remaining PCs did not show any important separation. Following the PCA-LDA cross-validation of all the epithelial spectra, as shown in Fig. 5, the classifier showed a sensitivity of 72%, a specificity of 88% (top panel), and an AUC value of 89% (bottom panel).

### Correlation of spectral features to biomolecular characteristics

**Fig. 6:**
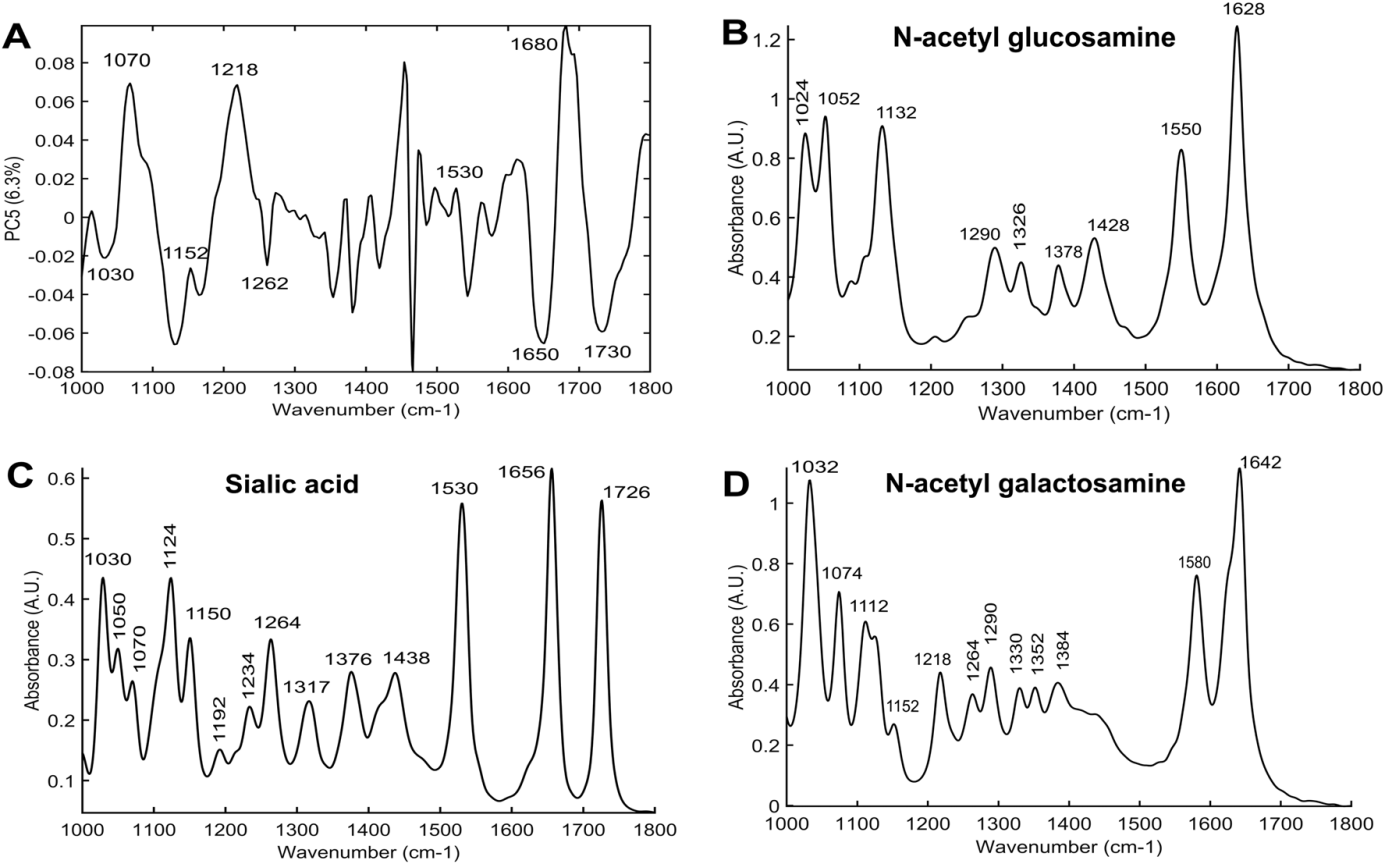
Spectral peak assignment to biomolecular features. Principal component loading 5 (**a**) showing features of sialic acid when compared with different glycan standards—N-acetylglucosamine (**b**), Sialic acid (**c**) and N-acetylgalactosamine (**d**).

In this study, an AUC performance of 95% when using mucus spectra and 89% for epithelial spectra was obtained in classifying cancer vs. non-cancer pathology. To achieve this, the IR spectral window from 1000 to 1800 cm^−^^1^ was used, which represents the global biomolecular composition found within the tissues, in contrast to label-based methods which are used for targeted detection. While the totality of PCs denote the global biomolecular variability, specific biomolecular components, or a combination of components could be denoted by individual PCs. Therefore, in addition to pathology classification, it was aimed to tentatively assign the PCs to biomolecular attributes. For the mucus-based classification, as expected the first few PC loadings (that show the highest explained variance) mostly indicated changes in the glycoprotein (1000–1300 cm^−1^ -glycan; 1500–1700 cm^−1^ -amide I and II of proteins) region (SI 1). Interestingly, PC5 showed changes further beyond the protein region at 1700–1800 cm^−1^ in addition to the glycan region (Fig. 6a), indicative of sialic acid molecules. To verify this, PC5 was compared with the glycan standards namely GalNAc, GlcNAc, and sialic acid, known to commonly vary in cancers, as shown in Fig. 6b–d. In comparison, PC5 spectral features could be assigned to sialic acid with characteristic peaks at 1030 cm^−1^, 1070, 1152, 1262, 1530, 1650, and 1726 cm^−1^. In particular, the peak at 1726 cm^−1^ appears to be specific of sialic acid which arises from ester vibrations and which is not present in the other glycan standards. Considering the epithelium PCs, the contributors for the classification appears to be distributed across the fingerprint region (1000–1800 cm^−1^) mostly arising from 1500 to 1700 cm^−1^ region (amide I and II of the protein region) (SI 2).

## Discussion

The main aim of this study was to test the suitability of mucus as a potential biological material for colorectal cancer screening/diagnosis. In this endeavor, IR hyperspectral images from colorectal tissues were obtained and subjected to statistical modeling.

In the first step, the IR spectral images were partitioned into their constituent histological features based on cluster analysis. Using K-means clustering, a clear segmentation of the important histological features, which in this case were mucus regions and the epithelial regions were obtained (Fig. 1). K-means clustering is a rapid way to digitally map associated biomolecular features within tissues. With respect to the two-stain H&E method, an advantage of cluster analysis is that it generates multiple digital stains that are color-coded and easily visualized. Furthermore, the biomolecular features are assigned specific coordinates on the digital maps, so they can be retrieved anytime for further analysis. In addition to delineating the important histological structures, it is also interesting to note a heterogeneity within the mucus of the tumoral sample as it is depicted by two clusters in Fig. 1f. This feature is sometimes observed in both normal and tumoral samples which is hard to appreciate using H&E staining. Whether such heterogeneity harbors biochemical changes that could be of diagnostic significance remains to be explored.

In the next step, classification models developed from the spectra extracted from the mucus and the epithelial regions were tested. Comparing the classification attributes of IR biochemical spectra from mucus and epithelium (Figs. 3 and 5), mucus showed a higher sensitivity of 96% for normal samples compared with 83% for cancer samples. In the opposite trend, the epithelium classification showed a lower sensitivity of 72% for normal samples but a higher sensitivity of 88% for cancer samples. Overall, the diagnostic value of the model was calculated by AUROC in which the mucus-based classification showed a higher AUC performance at 95% in comparison with epithelium at 89% in classifying cancer vs. non-cancer pathology. One of the possible reasons for higher AUC performance of mucus could be that the biochemical variations of glycans are spectroscopically more discernible. Mucus has been shown to undergo several alterations involving glycosylation, modified expression levels of antigens, reduced number and length of carbohydrate side chains and changes involving sialylation and sulfation [32]. On the other hand, the normal colon epithelial cells are known to be highly proliferative and have high mitotic rate similar to neoplastic cells. Therefore, the net variance of features such as nucleic acids, when compared with moderately differentiated tumors in which cellular proliferation is only slightly increased, these variations could be spectrally less apparent [29]. Nonetheless, in this study the AUC performance of epithelium is relatively high in comparison with other approaches.

Finally, a tentative assignment of spectral features that were significantly discriminant, to the biomolecular characteristics was sought. Comparing the commonly present glycans in mucus (Fig. 6) to the PCs, PC5 appears to indicate sialic acid contribution to the classification model particularly from the peak at 1726 cm^−1^ [33, 34].

Sialic acid that is present at the terminal ends of glycans in mucus, has been shown as a potential biomarker indicative of cancer using MS, surface enhanced Raman spectroscopy and immunohistochemistry [32, 35, 36]. So far, very few studies have been carried out using IR spectroscopy on mucus as a potential material for cancer diagnosis [27, 37]. These studies showed promising results where discrimination between normal and cancerous tissues was obtained based on mucin secondary structure changes, although attempts to link these changes to Muc2 and Muc5AC expression did not show any correlation. Importantly, there were also no significant differences in the glycan region/sialic acid specific components of mucus that we observed in this study. This observation therefore emphasizes the need to further understand the role of sialic acid and associated glycans in mucus, as potential indicators of cancer.

This work is a proof-of-concept study showing the possibility of exploiting mucus as a potential biological material using IR spectroscopy. In colorectal cancer screening and diagnosis, where excised polyps are scrutinized using histopathology, mucus could be an interesting adjunct that can provide early signs of neoplastic transformation that are phenotypically not expressed in histological sections. In addition to the cellular and tissue information, changes in the expression levels of acidic/neutral mucins, glycans, and glycosylation patterns could be of important diagnostic value.

All the aforementioned steps which are currently detected by chemical stains over long processing times, can be obtained via IR spectral imaging in a single measurement. In this way, both cellular, histological and mucus features can be analyzed at the same time.

In this study, mucus present within excised tissues has been studied using an imaging approach. The current workflow is a stainless-staining approach that uses spectroscopic imaging, digital de-paraffinization and cluster analysis for data segmentation and, PCA-LDA for pathology classification. As such, this ‘spectral histopathology’ diagnosis workflow can be rapid and automated to reduce human involvement at the same time complementing the current histopathological process.

It is important to note that at this point, the study is limited in terms of sample number. To strengthen and validate the findings, large-scale studies based on this concept are needed for better understanding the contributions of sialic acid and other glycans in mucus. Based on this concept and with the main goal of moving towards a ‘liquid biopsy’ approach, it is also crucial to study fresh mucus collected from patients. For this, a potential way forward could be to collect mucus as part of the FOBT screening [38, 39].

Subject to developing a collection protocol, studying mucus this way will avoid potential tissue fixation or embedding effects [40, 41]. Although, mucus collected this way may be more heterogeneous due to presence of fecal and other fibrous material, blood, bacteria etc, various purification steps could be used to test the extracted mucus either as a whole glycoprotein, or it could be broken down to separate the glycan and protein components. It is also possible to isolate individual glycan molecules such as sialic acid and quantify them using IR spectroscopy without the need for an imaging modality. Such studies would provide stronger basis for moving closer towards clinical applications. Furthermore, other data analytical streams encompassing Machine Learning algorithms such as Random Forests, Support Vector Machines, etc., may deliver improved outcomes over larger sample sizes, considering they are more difficult in providing information on the basis for their decision.

## Supplementary information

Supplemental Material
