# Supplementary material for: Characterization of colorectal mucus using infrared spectroscopy: a potential target for bowel cancer screening and diagnosis
Source: Lab Invest. 2020 Mar 20;100(8):1102–10. doi: 10.1038/s41374-020-0418-3 (PMC7374084; doi:10.1038/s41374-020-0418-3)
Supplement: Supplementary file 1 — Supplemental Material [file 41374_2020_418_MOESM1_ESM.pdf]

Sample Number	Sex	Age	Study code	Pathology	TNM	Stage	Sample Origin
1	M	48	Normal one	Adjacent normal	-	-	Whole section
2	F	71	Normal two	Adjacent normal	-	-	Whole section
3	F	71	Normal three	Adjacent normal	-	-	Whole section
4	M	72	Normal four	Adjacent normal	-	-	Whole section
5	M	45	Normal five	Adjacent normal	-	-	Whole section
6	M	67	Normal seven	Adjacent normal	-	-	Whole section
7	M	87	Normal ten	Adjacent normal	-	-	Whole section
8	M	87	Normal eleven	Adjacent normal	-	-	Whole section
9	M	61	TMA Normal	Adjacent normal	-	-	TMA
10	F	42	TMA Normal	Adjacent normal	-	-	TMA
11	M	48	Tumoral one	Moderately/poor differentiated adenocarcinoma	T3N1M0	III	Whole section
12	F	71	Tumoral two	Moderately differentiated adenocarcinoma	T3N1M0	IIa	Whole section
13	F	71	Tumoral three	Moderately differentiated adenocarcinoma	T2N1M0	IIIA	Whole section
14	M	72	Tumoral four	Moderately differentiated adenocarcinoma	T1N1M0	IIIA	Whole section
15	M	45	Tumoral five	Moderately differentiated adenocarcinoma	T3N1M0	III	Whole section
16	M	67	Turmoral seven	Moderately differentiated adenocarcinoma	T3N1M0	III	Whole section
17	M	48	TMA Tumoral	Mucinos adenocarcinoma	T3N1M0	III	TMA
18	F	55	TMA Tumoral	Mucinos adenocarcinoma	T3N1M0	III	TMA
19	M	70	TMA Tumoral	Mucinos adenocarcinoma	T3N0M0	IIA	TMA
20	M	36	TMA Tumoral	Mucinos adenocarcinoma	T2N0M0	I	TMA
21	M	60	TMA Tumoral	Mucinos adenocarcinoma	T3N0M0	IIA	TMA
22	M	39	TMA Tumoral	Mucinos adenocarcinoma	T3N0M0	IIA	TMA
23	F	40	TMA Tumoral	Mucinos adenocarcinoma	T3N0M0	IIA	TMA
24	F	69	TMA Tumoral	Mucinos adenocarcinoma	T3N0M0	IIA	TMA
25	F	44	TMA Tumoral	Mucinos adenocarcinoma	T3N0M0	IIA	TMA
26	M	74	TMA Tumoral	Mucinos adenocarcinoma	T3N1M0	III	TMA
			**Highlight indicates 'Same Patient'**				

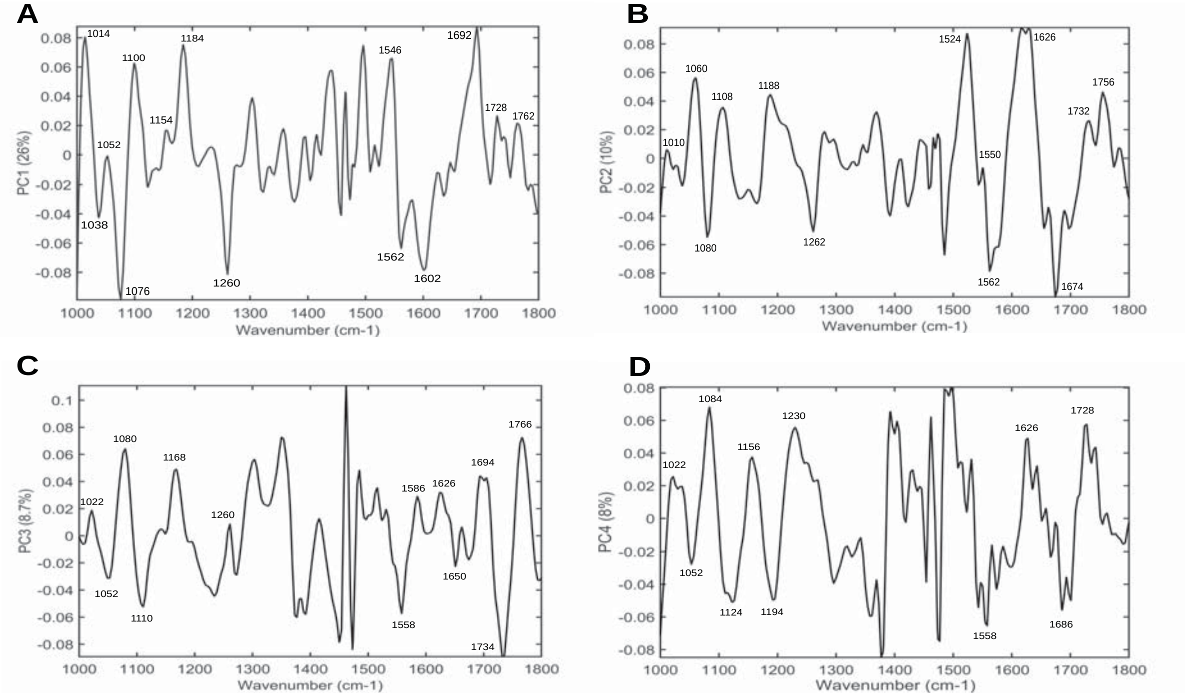
A
PC1 (26%)
1014
1100
1184
1154
1052
1038
1076
1260
1546
1692
1728
1762
1562
1602
Wavenumber (cm-1)
B
PC2 (10%)
1010
1060
1108
1188
1080
1262
1524
1626
1550
1562
1674
1732
1756
Wavenumber (cm-1)
C
PC3 (8.7%)
1022
1080
1168
1052
1110
1260
1586
1626
1694
1766
1650
1558
1734
Wavenumber (cm-1)
D
PC4 (8%)
1022
1084
1156
1230
1052
1124
1194
1626
1728
1558
1686
Wavenumber (cm-1)

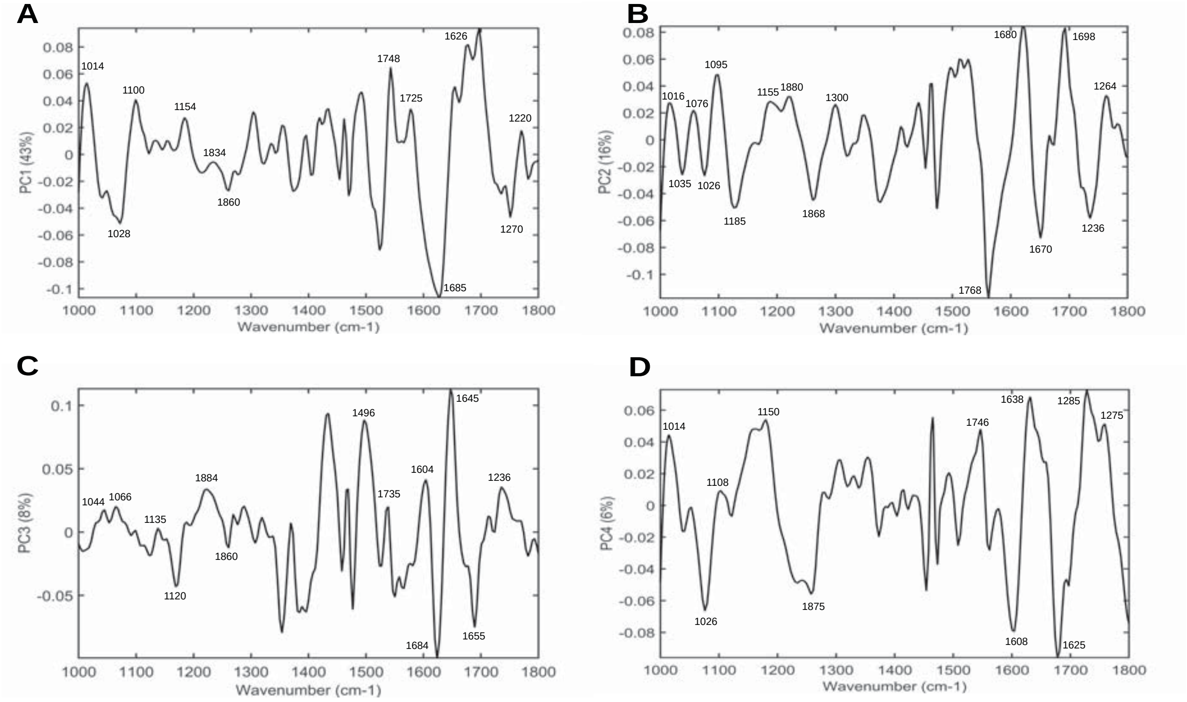

A
PC1 (43%)
Wavenumber (cm-1)
1014
1100
1154
1834
1860
1028
1748
1725
1626
1685
1220
1270
B
PC2 (16%)
Wavenumber (cm-1)
1016
1076
1095
1035
1026
1185
1155
1880
1868
1300
1768
1680
1670
1698
1264
1236
C
PC3 (8%)
Wavenumber (cm-1)
1044
1066
1135
1120
1884
1860
1496
1735
1604
1684
1645
1655
1236
D
PC4 (6%)
Wavenumber (cm-1)
1014
1108
1026
1150
1875
1746
1638
1608
1625
1285
1275

Supporting Information

Supporting information-Table 1: Clinical information

Clinical information of the samples including their origin, pathology and the TNM stage.

Supporting information 1: Principal component loadings of mucus spectra

The first few principal component loadings of mucus spectra show typical glycoprotein profile (1000-1300 cm^{-1} –glycan; 1500-1700 cm^{-1} -Amide I and II of proteins) with prominent spectral peaks highlighted.

Supporting information 2: Principal component loadings of epithelium spectra

The first few principal component loadings of epithelium spectra with prominent spectral peaks highlighted.
